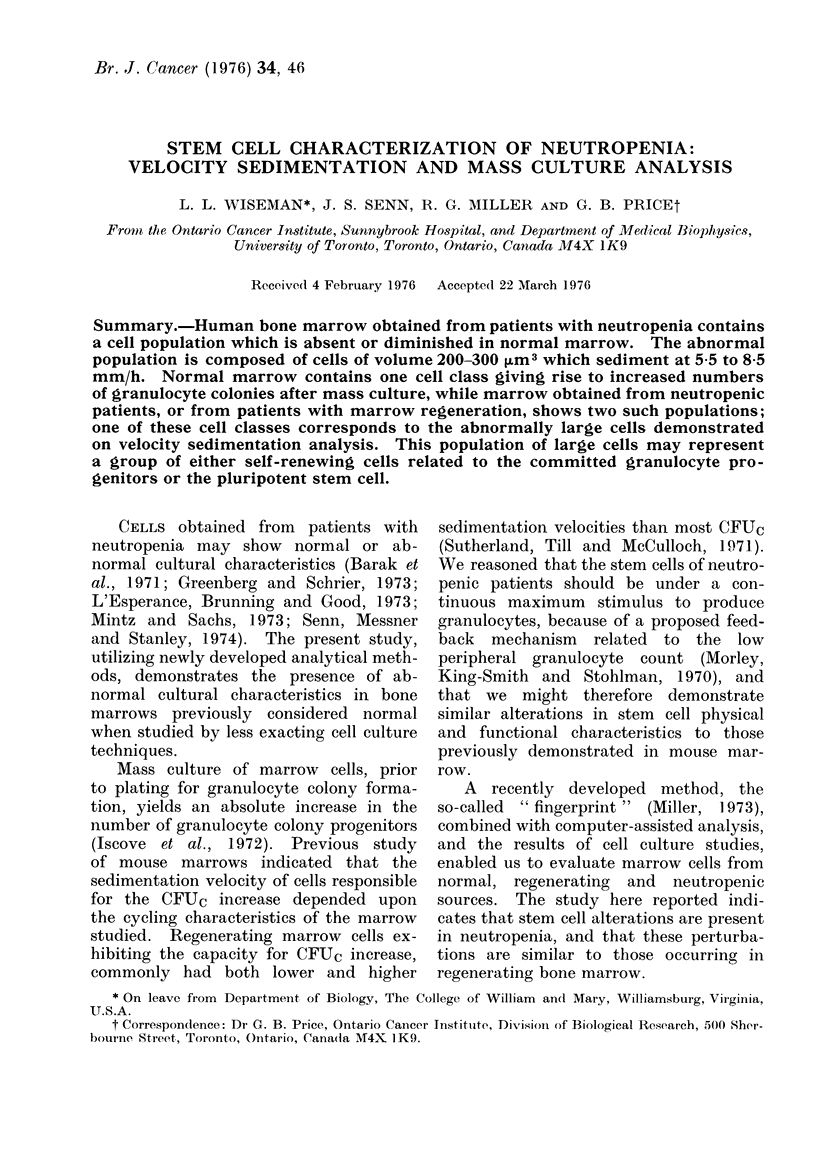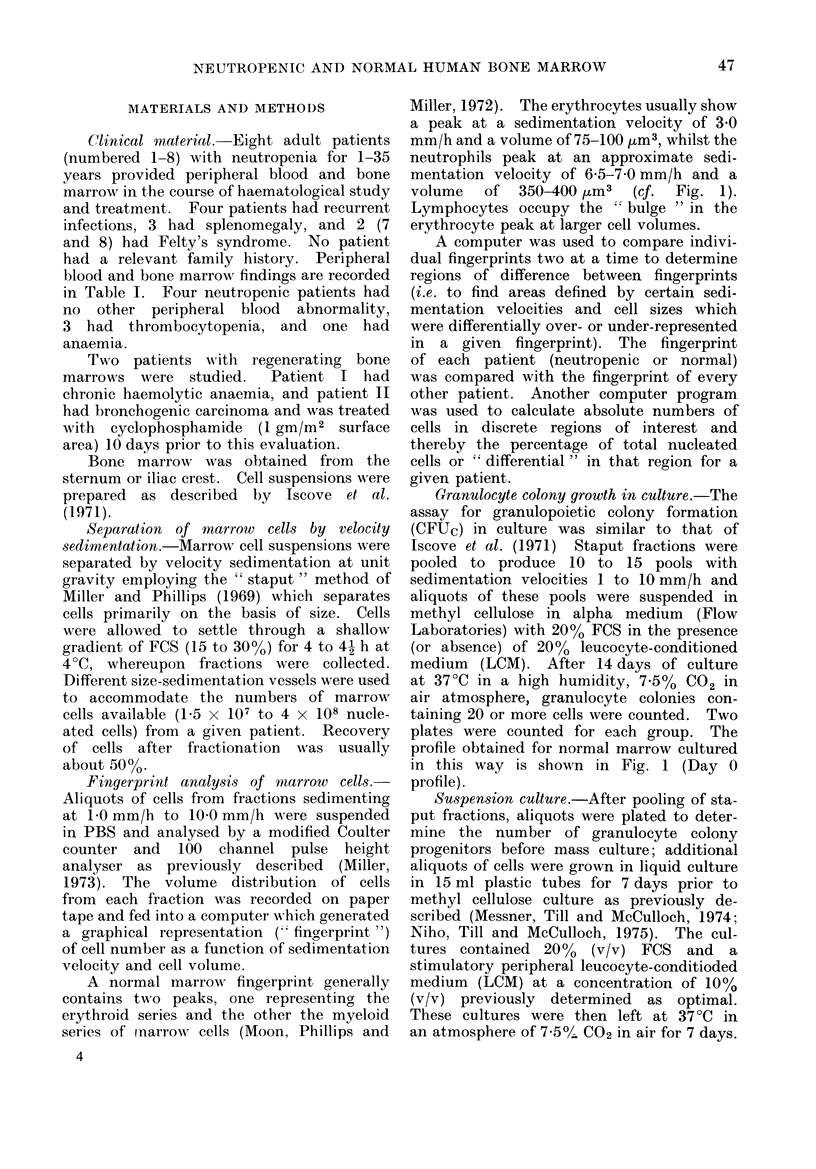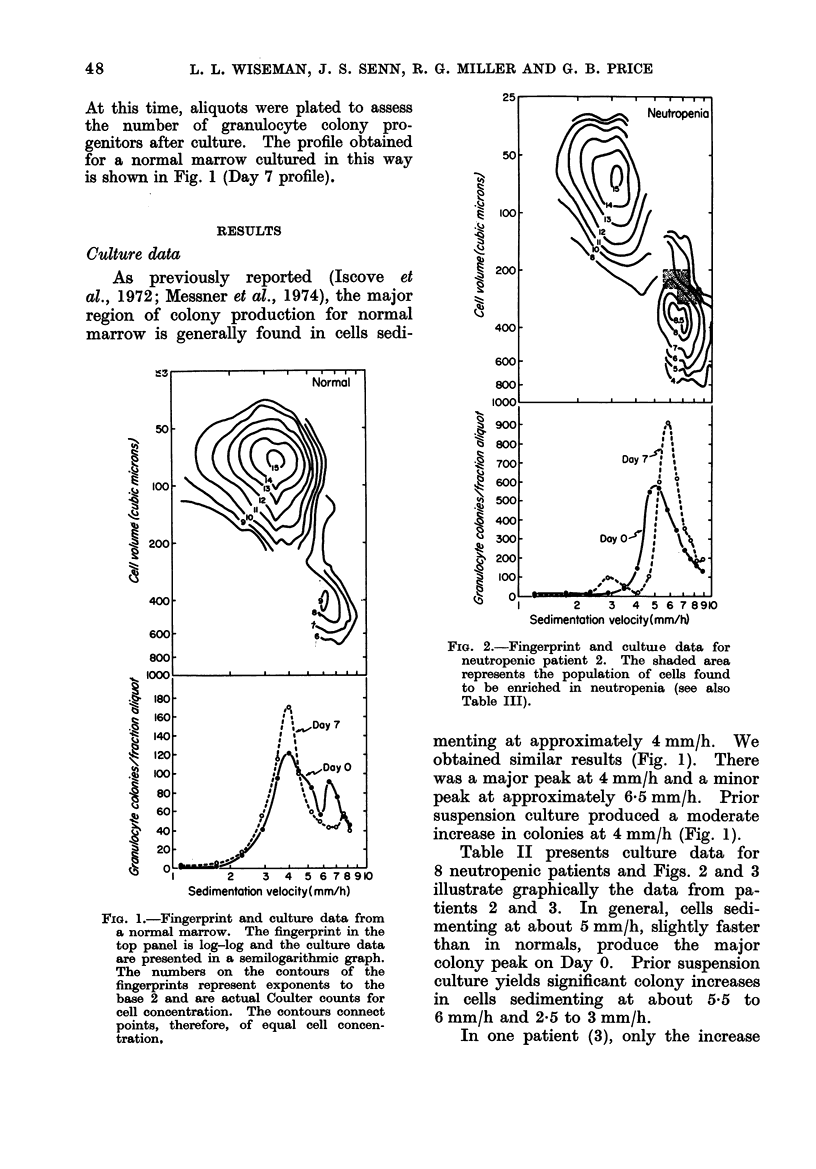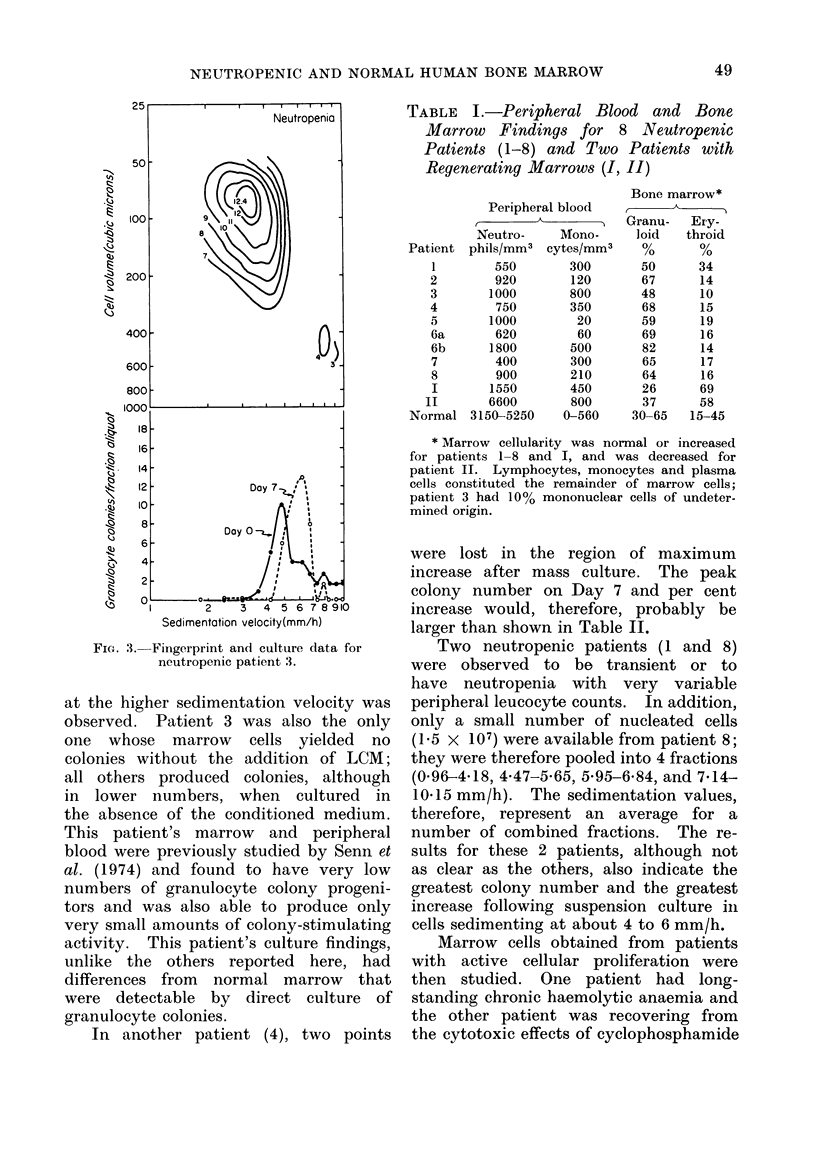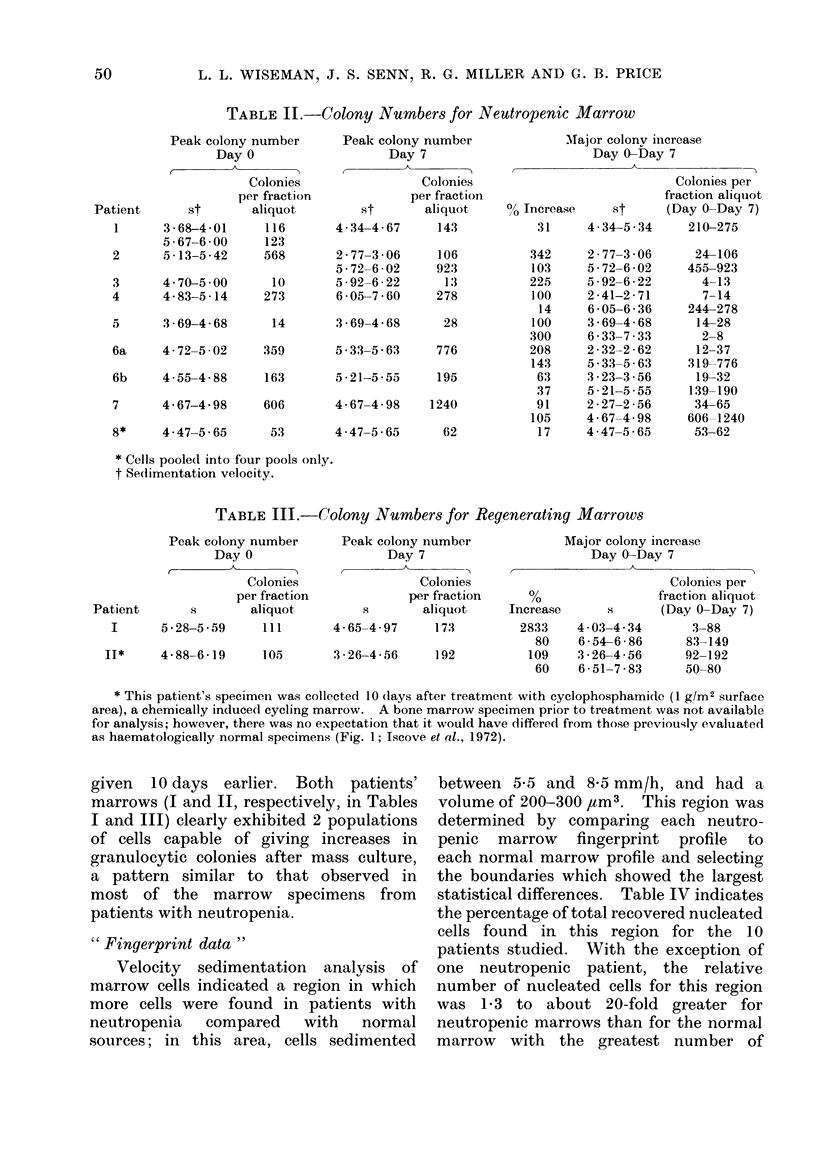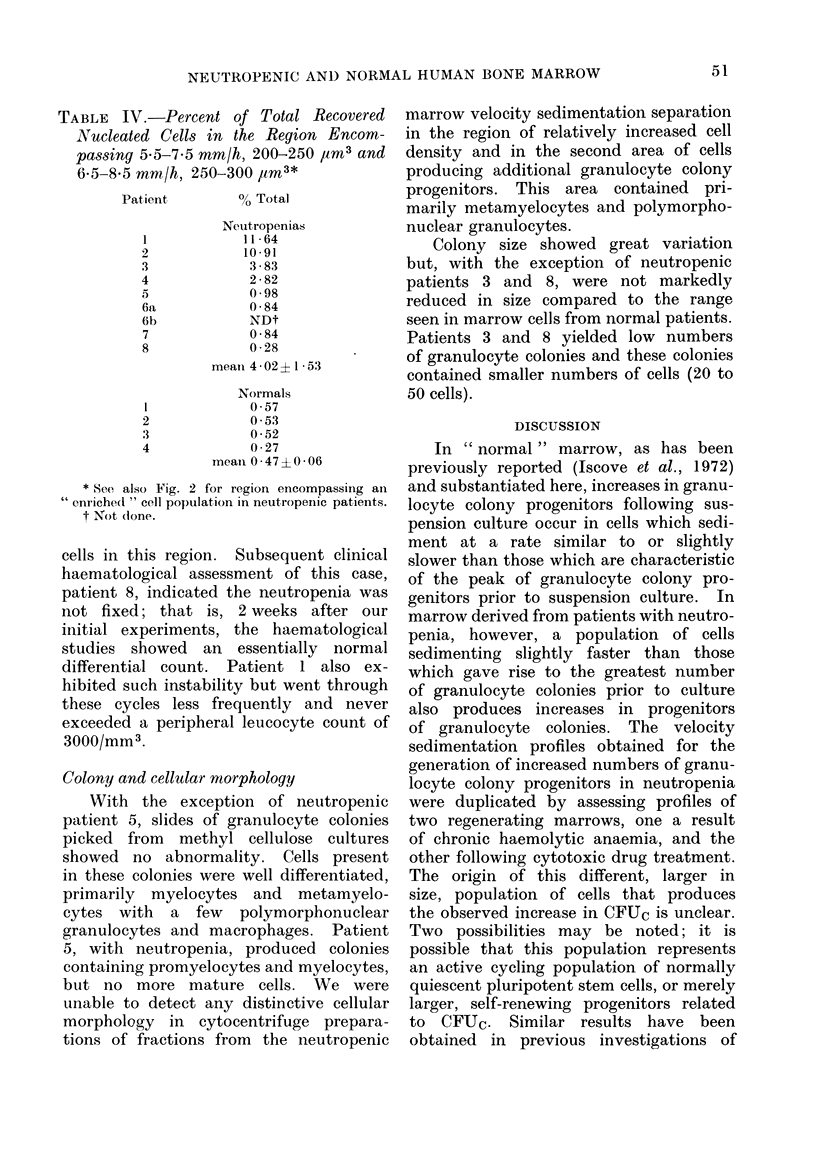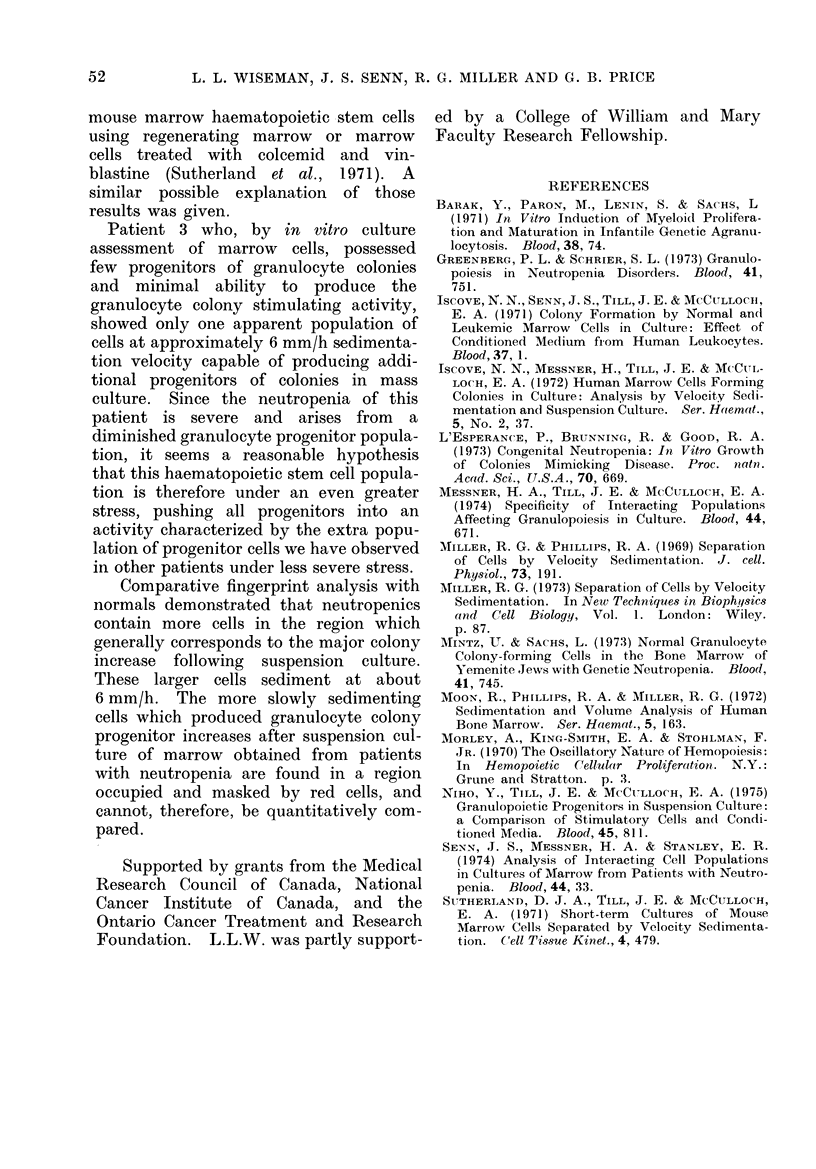# Stem cell characterization of neutropenia: velocity sedimentation and mass culture analysis.

**DOI:** 10.1038/bjc.1976.120

**Published:** 1976-07

**Authors:** L. L. Wiseman, J. S. Senn, R. G. Miller, G. B. Price

## Abstract

Human bone marrow obtained from patients with neutropenia contains a cell population which is absent or diminished in normal marrow. The abnormal population is composed of cells of volume 200-300 mum3 which sediment at 5-5 to 8-5 mm/h. Normal marrow contains one cell class giving rise to increased numbers of grnaulocyte colonies after mass culture, while marrow obtained from neutropenic patients, or from patients with marrow regeneration, shows two such populations; one of these cell classes corresponds to the abnormally large cells demonstrated on velocity sedimentation analysis. This population of large cells may represent a group of either self-renewing cells related to the committed granulocyte progenitors or the pluripotent stem cell.


*Br. J. Cancer* (1976) **34**, 46

### STEM CELL CHARACTERIZATION OF NEUTROPENIA: VELOCITY SEDIMENTATION AND MASS CULTURE ANALYSIS

L. L. WISEMAN*, J. S. SENN, R. G. MILLER AND G. B. PRICE†

*From the Ontario Cancer Institute, Sunnybrook Hospital, and Department of Medical Biophysics, University of Toronto, Toronto, Ontario, Canada M4X 1K9*

Received 4 February 1976 Accepted 22 March 1976

**Summary.—Human bone marrow obtained from patients with neutropenia contains a cell population which is absent or diminished in normal marrow. The abnormal population is composed of cells of volume 200–300 $\mu m^3$ which sediment at 5·5 to 8·5 mm/h. Normal marrow contains one cell class giving rise to increased numbers of granulocyte colonies after mass culture, while marrow obtained from neutropenic patients, or from patients with marrow regeneration, shows two such populations; one of these cell classes corresponds to the abnormally large cells demonstrated on velocity sedimentation analysis. This population of large cells may represent a group of either self-renewing cells related to the committed granulocyte progenitors or the pluripotent stem cell.**

CELLS obtained from patients with neutropenia may show normal or abnormal cultural characteristics (Barak *et al.*, 1971; Greenberg and Schrier, 1973; L'Esperance, Brunning and Good, 1973; Mintz and Sachs, 1973; Senn, Messner and Stanley, 1974). The present study, utilizing newly developed analytical methods, demonstrates the presence of abnormal cultural characteristics in bone marrows previously considered normal when studied by less exacting cell culture techniques.

Mass culture of marrow cells, prior to plating for granulocyte colony formation, yields an absolute increase in the number of granulocyte colony progenitors (Iscove *et al.*, 1972). Previous study of mouse marrows indicated that the sedimentation velocity of cells responsible for the $CFU_C$ increase depended upon the cycling characteristics of the marrow studied. Regenerating marrow cells exhibiting the capacity for $CFU_C$ increase, commonly had both lower and higher sedimentation velocities than most $CFU_C$ (Sutherland, Till and McCulloch, 1971). We reasoned that the stem cells of neutropenic patients should be under a continuous maximum stimulus to produce granulocytes, because of a proposed feedback mechanism related to the low peripheral granulocyte count (Morley, King-Smith and Stohlman, 1970), and that we might therefore demonstrate similar alterations in stem cell physical and functional characteristics to those previously demonstrated in mouse marrow.

A recently developed method, the so-called "fingerprint" (Miller, 1973), combined with computer-assisted analysis, and the results of cell culture studies, enabled us to evaluate marrow cells from normal, regenerating and neutropenic sources. The study here reported indicates that stem cell alterations are present in neutropenia, and that these perturbations are similar to those occurring in regenerating bone marrow.

* On leave from Department of Biology, The College of William and Mary, Williamsburg, Virginia, U.S.A.

† Correspondence: Dr G. B. Price, Ontario Cancer Institute, Division of Biological Research, 500 Sherbourne Street, Toronto, Ontario, Canada M4X 1K9.

### MATERIALS AND METHODS

*Clinical material.*—Eight adult patients (numbered 1–8) with neutropenia for 1–35 years provided peripheral blood and bone marrow in the course of haematological study and treatment. Four patients had recurrent infections, 3 had splenomegaly, and 2 (7 and 8) had Felty's syndrome. No patient had a relevant family history. Peripheral blood and bone marrow findings are recorded in Table I. Four neutropenic patients had no other peripheral blood abnormality, 3 had thrombocytopenia, and one had anaemia.

Two patients with regenerating bone marrows were studied. Patient I had chronic haemolytic anaemia, and patient II had bronchogenic carcinoma and was treated with cyclophosphamide (1 gm/m$^2$ surface area) 10 days prior to this evaluation.

Bone marrow was obtained from the sternum or iliac crest. Cell suspensions were prepared as described by Iscove *et al.* (1971).

*Separation of marrow cells by velocity sedimentation.*—Marrow cell suspensions were separated by velocity sedimentation at unit gravity employing the " staput " method of Miller and Phillips (1969) which separates cells primarily on the basis of size. Cells were allowed to settle through a shallow gradient of FCS (15 to 30%) for 4 to 4½ h at 4°C, whereupon fractions were collected. Different size-sedimentation vessels were used to accommodate the numbers of marrow cells available ($1\cdot5 \times 10^7$ to $4 \times 10^8$ nucleated cells) from a given patient. Recovery of cells after fractionation was usually about 50%.

*Fingerprint analysis of marrow cells.*—Aliquots of cells from fractions sedimenting at 1·0 mm/h to 10·0 mm/h were suspended in PBS and analysed by a modified Coulter counter and 100 channel pulse height analyser as previously described (Miller, 1973). The volume distribution of cells from each fraction was recorded on paper tape and fed into a computer which generated a graphical representation (" fingerprint ") of cell number as a function of sedimentation velocity and cell volume.

A normal marrow fingerprint generally contains two peaks, one representing the erythroid series and the other the myeloid series of marrow cells (Moon, Phillips and Miller, 1972). The erythrocytes usually show a peak at a sedimentation velocity of 3·0 mm/h and a volume of 75–100 $\mu$m$^3$, whilst the neutrophils peak at an approximate sedimentation velocity of 6·5–7·0 mm/h and a volume of 350–400 $\mu$m$^3$ (*cf.* Fig. 1). Lymphocytes occupy the " bulge " in the erythrocyte peak at larger cell volumes.

A computer was used to compare individual fingerprints two at a time to determine regions of difference between fingerprints (*i.e.* to find areas defined by certain sedimentation velocities and cell sizes which were differentially over- or under-represented in a given fingerprint). The fingerprint of each patient (neutropenic or normal) was compared with the fingerprint of every other patient. Another computer program was used to calculate absolute numbers of cells in discrete regions of interest and thereby the percentage of total nucleated cells or " differential " in that region for a given patient.

*Granulocyte colony growth in culture.*—The assay for granulopoietic colony formation (CFU$_C$) in culture was similar to that of Iscove *et al.* (1971) Staput fractions were pooled to produce 10 to 15 pools with sedimentation velocities 1 to 10 mm/h and aliquots of these pools were suspended in methyl cellulose in alpha medium (Flow Laboratories) with 20% FCS in the presence (or absence) of 20% leucocyte-conditioned medium (LCM). After 14 days of culture at 37°C in a high humidity, 7·5% $CO_2$ in air atmosphere, granulocyte colonies containing 20 or more cells were counted. Two plates were counted for each group. The profile obtained for normal marrow cultured in this way is shown in Fig. 1 (Day 0 profile).

*Suspension culture.*—After pooling of staput fractions, aliquots were plated to determine the number of granulocyte colony progenitors before mass culture; additional aliquots of cells were grown in liquid culture in 15 ml plastic tubes for 7 days prior to methyl cellulose culture as previously described (Messner, Till and McCulloch, 1974; Niho, Till and McCulloch, 1975). The cultures contained 20% (v/v) FCS and a stimulatory peripheral leucocyte-conditioded medium (LCM) at a concentration of 10% (v/v) previously determined as optimal. These cultures were then left at 37°C in an atmosphere of 7·5% $CO_2$ in air for 7 days.

At this time, aliquots were plated to assess the number of granulocyte colony progenitors after culture. The profile obtained for a normal marrow cultured in this way is shown in Fig. 1 (Day 7 profile).

### RESULTS

### *Culture data*

As previously reported (Iscove *et al.*, 1972; Messner *et al.*, 1974), the major region of colony production for normal marrow is generally found in cells sedimenting at approximately 4 mm/h. We obtained similar results (Fig. 1). There was a major peak at 4 mm/h and a minor peak at approximately 6·5 mm/h. Prior suspension culture produced a moderate increase in colonies at 4 mm/h (Fig. 1).

FIG. 1.—Fingerprint and culture data from a normal marrow. The fingerprint in the top panel is log–log and the culture data are presented in a semilogarithmic graph. The numbers on the contours of the fingerprints represent exponents to the base 2 and are actual Coulter counts for cell concentration. The contours connect points, therefore, of equal cell concentration.

FIG. 2.—Fingerprint and cultue data for neutropenic patient 2. The shaded area represents the population of cells found to be enriched in neutropenia (see also Table III).

Table II presents culture data for 8 neutropenic patients and Figs. 2 and 3 illustrate graphically the data from patients 2 and 3. In general, cells sedimenting at about 5 mm/h, slightly faster than in normals, produce the major colony peak on Day 0. Prior suspension culture yields significant colony increases in cells sedimenting at about 5·5 to 6 mm/h and 2·5 to 3 mm/h.

In one patient (3), only the increase

FIG. 3.—Fingerprint and culture data for neutropenic patient 3.

at the higher sedimentation velocity was observed. Patient 3 was also the only one whose marrow cells yielded no colonies without the addition of LCM; all others produced colonies, although in lower numbers, when cultured in the absence of the conditioned medium. This patient's marrow and peripheral blood were previously studied by Senn *et al.* (1974) and found to have very low numbers of granulocyte colony progenitors and was also able to produce only very small amounts of colony-stimulating activity. This patient's culture findings, unlike the others reported here, had differences from normal marrow that were detectable by direct culture of granulocyte colonies.

In another patient (4), two points were lost in the region of maximum increase after mass culture. The peak colony number on Day 7 and per cent increase would, therefore, probably be larger than shown in Table II.

TABLE I.—*Peripheral Blood and Bone Marrow Findings for* 8 *Neutropenic Patients* (1–8) *and Two Patients with Regenerating Marrows* (*I*, *II*)

| | Peripheral blood | | Bone marrow* | |
|---|---|---|---|---|
| Patient | Neutrophils/mm³ | Monocytes/mm³ | Granuloid % | Erythroid % |
| 1 | 550 | 300 | 50 | 34 |
| 2 | 920 | 120 | 67 | 14 |
| 3 | 1000 | 800 | 48 | 10 |
| 4 | 750 | 350 | 68 | 15 |
| 5 | 1000 | 20 | 59 | 19 |
| 6a | 620 | 60 | 69 | 16 |
| 6b | 1800 | 500 | 82 | 14 |
| 7 | 400 | 300 | 65 | 17 |
| 8 | 900 | 210 | 64 | 16 |
| I | 1550 | 450 | 26 | 69 |
| II | 6600 | 800 | 37 | 58 |
| Normal | 3150–5250 | 0–560 | 30–65 | 15–45 |

* Marrow cellularity was normal or increased for patients 1–8 and I, and was decreased for patient II. Lymphocytes, monocytes and plasma cells constituted the remainder of marrow cells; patient 3 had 10% mononuclear cells of undetermined origin.

Two neutropenic patients (1 and 8) were observed to be transient or to have neutropenia with very variable peripheral leucocyte counts. In addition, only a small number of nucleated cells ($1 \cdot 5 \times 10^7$) were available from patient 8; they were therefore pooled into 4 fractions (0·96–4·18, 4·47–5·65, 5·95–6·84, and 7·14–10·15 mm/h). The sedimentation values, therefore, represent an average for a number of combined fractions. The results for these 2 patients, although not as clear as the others, also indicate the greatest colony number and the greatest increase following suspension culture in cells sedimenting at about 4 to 6 mm/h.

Marrow cells obtained from patients with active cellular proliferation were then studied. One patient had long-standing chronic haemolytic anaemia and the other patient was recovering from the cytotoxic effects of cyclophosphamide

TABLE II.—*Colony Numbers for Neutropenic Marrow*

| | Peak colony number Day 0 | | Peak colony number Day 7 | | Major colony increase Day 0–Day 7 | | |
|---|---|---|---|---|---|---|---|
| Patient | s† | Colonies per fraction aliquot | s† | Colonies per fraction aliquot | % Increase | s† | Colonies per fraction aliquot (Day 0–Day 7) |
| 1 | 3·68–4·01 | 116 | 4·34–4·67 | 143 | 31 | 4·34–5·34 | 210–275 |
| | 5·67–6·00 | 123 | | | | | |
| 2 | 5·13–5·42 | 568 | 2·77–3·06 | 106 | 342 | 2·77–3·06 | 24–106 |
| | | | 5·72–6·02 | 923 | 103 | 5·72–6·02 | 455–923 |
| 3 | 4·70–5·00 | 10 | 5·92–6·22 | 13 | 225 | 5·92–6·22 | 4–13 |
| 4 | 4·83–5·14 | 273 | 6·05–7·60 | 278 | 100 | 2·41–2·71 | 7–14 |
| | | | | | 14 | 6·05–6·36 | 244–278 |
| 5 | 3·69–4·68 | 14 | 3·69–4·68 | 28 | 100 | 3·69–4·68 | 14–28 |
| | | | | | 300 | 6·33–7·33 | 2–8 |
| 6a | 4·72–5·02 | 359 | 5·33–5·63 | 776 | 208 | 2·32–2·62 | 12–37 |
| | | | | | 143 | 5·33–5·63 | 319–776 |
| 6b | 4·55–4·88 | 163 | 5·21–5·55 | 195 | 63 | 3·23–3·56 | 19–32 |
| | | | | | 37 | 5·21–5·55 | 139–190 |
| 7 | 4·67–4·98 | 606 | 4·67–4·98 | 1240 | 91 | 2·27–2·56 | 34–65 |
| | | | | | 105 | 4·67–4·98 | 606–1240 |
| 8* | 4·47–5·65 | 53 | 4·47–5·65 | 62 | 17 | 4·47–5·65 | 53–62 |

* Cells pooled into four pools only.
† Sedimentation velocity.

TABLE III.—*Colony Numbers for Regenerating Marrows*

| | Peak colony number Day 0 | | Peak colony number Day 7 | | Major colony increase Day 0–Day 7 | | |
|---|---|---|---|---|---|---|---|
| Patient | s | Colonies per fraction aliquot | s | Colonies per fraction aliquot | % Increase | s | Colonies per fraction aliquot (Day 0–Day 7) |
| I | 5·28–5·59 | 111 | 4·65–4·97 | 173 | 2833 | 4·03–4·34 | 3–88 |
| | | | | | 80 | 6·54–6·86 | 83–149 |
| II* | 4·88–6·19 | 105 | 3·26–4·56 | 192 | 109 | 3·26–4·56 | 92–192 |
| | | | | | 60 | 6·51–7·83 | 50–80 |

* This patient's specimen was collected 10 days after treatment with cyclophosphamide (1 g/m$^2$ surface area), a chemically induced cycling marrow. A bone marrow specimen prior to treatment was not available for analysis; however, there was no expectation that it would have differed from those previously evaluated as haematologically normal specimens (Fig. 1; Iscove *et al.*, 1972).

given 10 days earlier. Both patients' marrows (I and II, respectively, in Tables I and III) clearly exhibited 2 populations of cells capable of giving increases in granulocytic colonies after mass culture, a pattern similar to that observed in most of the marrow specimens from patients with neutropenia.

"*Fingerprint data*"

Velocity sedimentation analysis of marrow cells indicated a region in which more cells were found in patients with neutropenia compared with normal sources; in this area, cells sedimented between 5·5 and 8·5 mm/h, and had a volume of 200–300 $\mu m^3$. This region was determined by comparing each neutropenic marrow fingerprint profile to each normal marrow profile and selecting the boundaries which showed the largest statistical differences. Table IV indicates the percentage of total recovered nucleated cells found in this region for the 10 patients studied. With the exception of one neutropenic patient, the relative number of nucleated cells for this region was 1·3 to about 20-fold greater for neutropenic marrows than for the normal marrow with the greatest number of

TABLE IV.—*Percent of Total Recovered Nucleated Cells in the Region Encompassing* 5·5–7·5 *mm/h*, 200–250 $\mu m^3$ *and* 6·5–8·5 *mm/h*, 250–300 $\mu m^3$*

| Patient | % Total |
|---|---|
| | Neutropenias |
| 1 | 11·64 |
| 2 | 10·91 |
| 3 | 3·83 |
| 4 | 2·82 |
| 5 | 0·98 |
| 6a | 0·84 |
| 6b | ND† |
| 7 | 0·84 |
| 8 | 0·28 |
| | mean 4·02 ± 1·53 |
| | Normals |
| 1 | 0·57 |
| 2 | 0·53 |
| 3 | 0·52 |
| 4 | 0·27 |
| | mean 0·47 ± 0·06 |

* See also Fig. 2 for region encompassing an " enriched " cell population in neutropenic patients.
† Not done.

cells in this region. Subsequent clinical haematological assessment of this case, patient 8, indicated the neutropenia was not fixed; that is, 2 weeks after our initial experiments, the haematological studies showed an essentially normal differential count. Patient 1 also exhibited such instability but went through these cycles less frequently and never exceeded a peripheral leucocyte count of 3000/mm$^3$.

*Colony and cellular morphology*

With the exception of neutropenic patient 5, slides of granulocyte colonies picked from methyl cellulose cultures showed no abnormality. Cells present in these colonies were well differentiated, primarily myelocytes and metamyelocytes with a few polymorphonuclear granulocytes and macrophages. Patient 5, with neutropenia, produced colonies containing promyelocytes and myelocytes, but no more mature cells. We were unable to detect any distinctive cellular morphology in cytocentrifuge preparations of fractions from the neutropenic marrow velocity sedimentation separation in the region of relatively increased cell density and in the second area of cells producing additional granulocyte colony progenitors. This area contained primarily metamyelocytes and polymorphonuclear granulocytes.

Colony size showed great variation but, with the exception of neutropenic patients 3 and 8, were not markedly reduced in size compared to the range seen in marrow cells from normal patients. Patients 3 and 8 yielded low numbers of granulocyte colonies and these colonies contained smaller numbers of cells (20 to 50 cells).

DISCUSSION

In " normal " marrow, as has been previously reported (Iscove *et al.*, 1972) and substantiated here, increases in granulocyte colony progenitors following suspension culture occur in cells which sediment at a rate similar to or slightly slower than those which are characteristic of the peak of granulocyte colony progenitors prior to suspension culture. In marrow derived from patients with neutropenia, however, a population of cells sedimenting slightly faster than those which gave rise to the greatest number of granulocyte colonies prior to culture also produces increases in progenitors of granulocyte colonies. The velocity sedimentation profiles obtained for the generation of increased numbers of granulocyte colony progenitors in neutropenia were duplicated by assessing profiles of two regenerating marrows, one a result of chronic haemolytic anaemia, and the other following cytotoxic drug treatment. The origin of this different, larger in size, population of cells that produces the observed increase in $CFU_C$ is unclear. Two possibilities may be noted; it is possible that this population represents an active cycling population of normally quiescent pluripotent stem cells, or merely larger, self-renewing progenitors related to $CFU_C$. Similar results have been obtained in previous investigations of mouse marrow haematopoietic stem cells using regenerating marrow or marrow cells treated with colcemid and vinblastine (Sutherland *et al.*, 1971). A similar possible explanation of those results was given.

Patient 3 who, by *in vitro* culture assessment of marrow cells, possessed few progenitors of granulocyte colonies and minimal ability to produce the granulocyte colony stimulating activity, showed only one apparent population of cells at approximately 6 mm/h sedimentation velocity capable of producing additional progenitors of colonies in mass culture. Since the neutropenia of this patient is severe and arises from a diminished granulocyte progenitor population, it seems a reasonable hypothesis that this haematopoietic stem cell population is therefore under an even greater stress, pushing all progenitors into an activity characterized by the extra population of progenitor cells we have observed in other patients under less severe stress.

Comparative fingerprint analysis with normals demonstrated that neutropenics contain more cells in the region which generally corresponds to the major colony increase following suspension culture. These larger cells sediment at about 6 mm/h. The more slowly sedimenting cells which produced granulocyte colony progenitor increases after suspension culture of marrow obtained from patients with neutropenia are found in a region occupied and masked by red cells, and cannot, therefore, be quantitatively compared.

Supported by grants from the Medical Research Council of Canada, National Cancer Institute of Canada, and the Ontario Cancer Treatment and Research Foundation. L.L.W. was partly supported by a College of William and Mary Faculty Research Fellowship.

### REFERENCES

Barak, Y., Paron, M., Lenin, S. & Sachs, L (1971) *In Vitro* Induction of Myeloid Proliferation and Maturation in Infantile Genetic Agranulocytosis. *Blood*, **38**, 74.

Greenberg, P. L. & Schrier, S. L. (1973) Granulopoiesis in Neutropenia Disorders. *Blood*, **41**, 751.

Iscove, N. N., Senn, J. S., Till, J. E. & McCulloch, E. A. (1971) Colony Formation by Normal and Leukemic Marrow Cells in Culture: Effect of Conditioned Medium from Human Leukocytes. *Blood*, **37**, 1.

Iscove, N. N., Messner, H., Till, J. E. & McCulloch, E. A. (1972) Human Marrow Cells Forming Colonies in Culture: Analysis by Velocity Sedimentation and Suspension Culture. *Ser. Haemat.*, **5**, No. 2, 37.

L'Esperance, P., Brunning, R. & Good, R. A. (1973) Congenital Neutropenia: *In Vitro* Growth of Colonies Mimicking Disease. *Proc. natn. Acad. Sci., U.S.A.*, **70**, 669.

Messner, H. A., Till, J. E. & McCulloch, E. A. (1974) Specificity of Interacting Populations Affecting Granulopoiesis in Culture. *Blood*, **44**, 671.

Miller, R. G. & Phillips, R. A. (1969) Separation of Cells by Velocity Sedimentation. *J. cell. Physiol.*, **73**, 191.

Miller, R. G. (1973) Separation of Cells by Velocity Sedimentation. In *New Techniques in Biophysics and Cell Biology*, Vol. 1. London: Wiley. p. 87.

Mintz, U. & Sachs, L. (1973) Normal Granulocyte Colony-forming Cells in the Bone Marrow of Yemenite Jews with Genetic Neutropenia. *Blood*, **41**, 745.

Moon, R., Phillips, R. A. & Miller, R. G. (1972) Sedimentation and Volume Analysis of Human Bone Marrow. *Ser. Haemat.*, **5**, 163.

Morley, A., King-Smith, E. A. & Stohlman, F. Jr. (1970) The Oscillatory Nature of Hemopoiesis: In *Hemopoietic Cellular Proliferation*. N.Y.: Grune and Stratton. p. 3.

Niho, Y., Till, J. E. & McCulloch, E. A. (1975) Granulopoietic Progenitors in Suspension Culture: a Comparison of Stimulatory Cells and Conditioned Media. *Blood*, **45**, 811.

Senn, J. S., Messner, H. A. & Stanley, E. R. (1974) Analysis of Interacting Cell Populations in Cultures of Marrow from Patients with Neutropenia. *Blood*, **44**, 33.

Sutherland, D. J. A., Till, J. E. & McCulloch, E. A. (1971) Short-term Cultures of Mouse Marrow Cells Separated by Velocity Sedimentation. *Cell Tissue Kinet.*, **4**, 479.